# Embracing Complexity: Yeast Evolution Experiments Featuring Standing Genetic Variation

**DOI:** 10.1007/s00239-023-10094-4

**Published:** 2023-02-08

**Authors:** Molly K. Burke

**Affiliations:** 1 Department of Integrative Biology, Oregon State University, Corvallis, OR 97333, USA

**Keywords:** Yeast, Experimental evolution, Standing genetic variation, Outcrossing

## Abstract

The yeast *Saccharomyces cerevisiae* has a long and esteemed history as a model system for laboratory selection experiments. The majority of yeast evolution experiments begin with an isogenic ancestor, impose selection as cells divide asexually, and track mutations that arise and accumulate over time. Within the last decade, the popularity of *S. cerevisiae* as a model system for exploring the evolution of standing genetic variation has grown considerably. As a facultatively sexual microbe, it is possible to initiate experiments with populations that harbor diversity and also to maintain that diversity by promoting sexual recombination as the experiment progresses. These experimental choices expand the scope of evolutionary hypotheses that can be tested with yeast. And, in this review, I argue that yeast is one of the best model systems for testing such hypotheses relevant to eukaryotic species. Here, I compile a list of yeast evolution experiments that involve standing genetic variation, initially and/or by implementing protocols that induce sexual recombination in evolving populations. I also provide an overview of experimental methods required to set up such an experiment and discuss the unique challenges that arise in this type of research. Throughout the article, I emphasize the best practices emerging from this small but growing niche of the literature.

## Introduction

For long-term evolutionary experiments, microbes are the gold standard model system for many reasons. Investigators can easily create and maintain large populations of bacteria or fungi, impose a selective environment for hundreds or thousands of generations, and track the fitness and genome changes that result in real time. A feature of most microbial systems that is difficult or impossible to achieve in higher eukaryotic populations is their total isogenicity; through single-cell bottlenecking one can produce a clonal lineage in which no variation is initially segregating. This provides a useful platform for addressing many questions that have historically fascinated experimental evolutionists about the mode and tempo of adaptive evolution. In an initially isogenic population, adaptation should proceed by the sequential fixation of de novo mutations that confer some evolutionary benefit, although this is complicated by hitchhiking and clonal interference. Identifying the location of these mutations often reveals novel insight into the genetic basis underlying specific traits, and identifying the order of these fixation events often reveals novel insight into evolutionary dynamics, such as the role of genetic parallelism (e.g., Tenaillon et al. 2012), historical contingency (e.g., Toprak et al. 2011), and epistasis (particularly diminishing-returns epistasis, e.g., Jerison & Desai 2015; Wang et al. 2016) in microbial adaptation.

Experimental evolutionists working with eukaryotes, typically *Drosophila*, face limitations related to the life history of their systems, and these limitations have led to important foundational discoveries about the genetic basis of adaptation. For example, selection experiments with eukaryotic systems generally support the idea that pre-existing variation rapidly and primarily drives adaptation (Burke et al. 2010; Graves et al. 2017; Bargi et al. 2020; Rêgo et al. 2020; O’Connor et al. 2021, among others). That is to say, the sequential fixation of beneficial mutations does not appear to drive adaptation when populations have abundant genetic variation upon which natural selection can act. But, these experiments typically feature systems in which the population size limits the number of de novo mutations per generation (i.e., 2Nμ is limited by N). Furthermore, in these systems the total number of generations is often fewer than 100, and as a result de novo mutations have little time to reach intermediate frequencies unless they have large (> 1%) selection coefficients. Thus, there has been growing interest in building model microbial systems for selection experiments that are capable of harboring and maintaining standing genetic variation. In theory, such a system would combine the practical features of working with a microbe, such as the ability to create experimental populations with large effective population sizes in which de novo beneficial mutations are likely to arise in a single generation, with the ideological features of working with a higher eukaryote, such as their applicability to other organisms, including humans.

So, which microbial systems are best suited for studying the evolution of standing genetic variation? In theory, a bacterial population could be established that harbors diversity, either by the intentional combination of clonal lineages into a pool or using a mutator strain as the ancestor. Such a variable bacterial population could then adapt to a novel environment via either selection on de novo beneficial mutations or pre-existing variants. But, in the absence of sexual recombination, the clonal lineage bearing the most beneficial genotype will come to dominate the population and exclude all other lineages. At that point, all variation are erased, and the population effectively resets; in other words, isogenicity returns. This type of adaptive walk is fundamentally different than what occurs in sexually reproducing populations, in which recombination uncouples individual alleles responding to selection from the rest of the genome (reviewed by Burke 2012). In sexual populations, variation is continuously maintained such that selection impacts small chromosomal regions, while the rest of the genome evolves neutrally. Therefore, a microorganism with a sexual life history is best suited for experimental evolution studies where the focus is on standing genetic variation. Nematodes, particularly *Caenorhabditis*, are an obvious choice, due to their ability to outcross (reviewed by Teotonio et al. 2017). A significant resource is the *Caenorhabditis elegans* Multiparent Experimental Evolution (CEMEE; Noble et al. 2017); this population is created by crossing 16 founder lines, harbors considerable standing genetic variation, and has been used as the ancestor for evolution experiments (e.g., Theologidis et al. 2014; Guzella et al. 2018). The benefits of using a metazoan model notwithstanding, some experimental design parameters cannot be optimally achieved in nematode populations, due to their relatively long (for a microorganism) generation time, and the need to culture populations on solid media limits population size. *Saccharomyces cerevisiae* has emerged as perhaps the best microbial system for addressing questions about the evolution of standing genetic variation. As a facultatively sexual budding yeast, *S. cerevisiae* checks nearly every box that is desirable for such work: it has rapid generational turnover, it is easy to maintain in populations of hundreds of thousands of individual cells cultured in either liquid or solid media, it is cryopreservable, it has unparalleled genomic resources, and benchwork can be easily automated for high throughput. This review examines *S. cerevisiae* evolution experiments that feature standing genetic variation, discusses limitations and experimental design considerations relevant to such work, and aims to synthesize the best practices emerging from the literature.

## Experimental Design

*S. cerevisiae* is especially well suited for experimental evolutionary studies involving standing genetic variation for many reasons. First, the species itself harbors a great deal of genetic diversity, due to its broad ecological range, and long history of domestication (e.g., Fay & Benavides 2005). Liti et al. (2009) were the first to characterize the population genetics of the species and identified > 200,000 high-quality SNPs (roughly 2% of the nuclear genome), as well as > 14,000 small indels, segregating across a collection of 38 strains isolated from natural, industrial, and clinical sources. They also identified five primary lineages generally corresponding to different regions of origin: Malaysia, West Africa, North America, sake and related fermentation strains, and European wine strains. Another key observation of this survey was that linkage disequilibrium decays rapidly in the species, with a half-maximum at < 3 kb. This implies high levels of recombination and outcrossing in the species, at least relative to the related species *S. paradoxus*. Peter et al. (2018) expanded our depth of genomic knowledge of the species by sequencing over 1000 *S. cerevisiae* isolates. They detected approximately 10X more variation than Liti et al. (2009) in the nuclear genome across this large collection of strains, with most SNPs observed at very low frequencies. Peter et al. (2018) also observed considerable variation in ploidy and aneuploidy across the species, although about 87% of surveyed isolates were classified as diploid; diploidy appears to be the state associated with highest fitness for most strains. Surveys of phenotypic variation across *S. cerevisiae* isolates reveal substantial and continuous levels of phenotypic variation, which is consistent with their apparent genomic complexity and also implies polygenicity for many yeast traits (e.g., Warringer et al. 2011, Bergstrom et al. 2014, Peter et al. 2018). This impressive species-level diversity of a model organism has long been exploited in the context of quantitative trait locus (QTL) mapping (reviewed by Liti and Louis 2012). Traditional QTL mapping approaches involve crossing two or more divergent strains and then genotyping and phenotyping the segregants in order to carry out linkage mapping. More recently, this diversity has also been leveraged, to varying degrees, for experimental evolution work. Table 1 provides a list of yeast evolution experiments that feature standing genetic variation and/or sex in prominent ways.

**Fig. 1:**
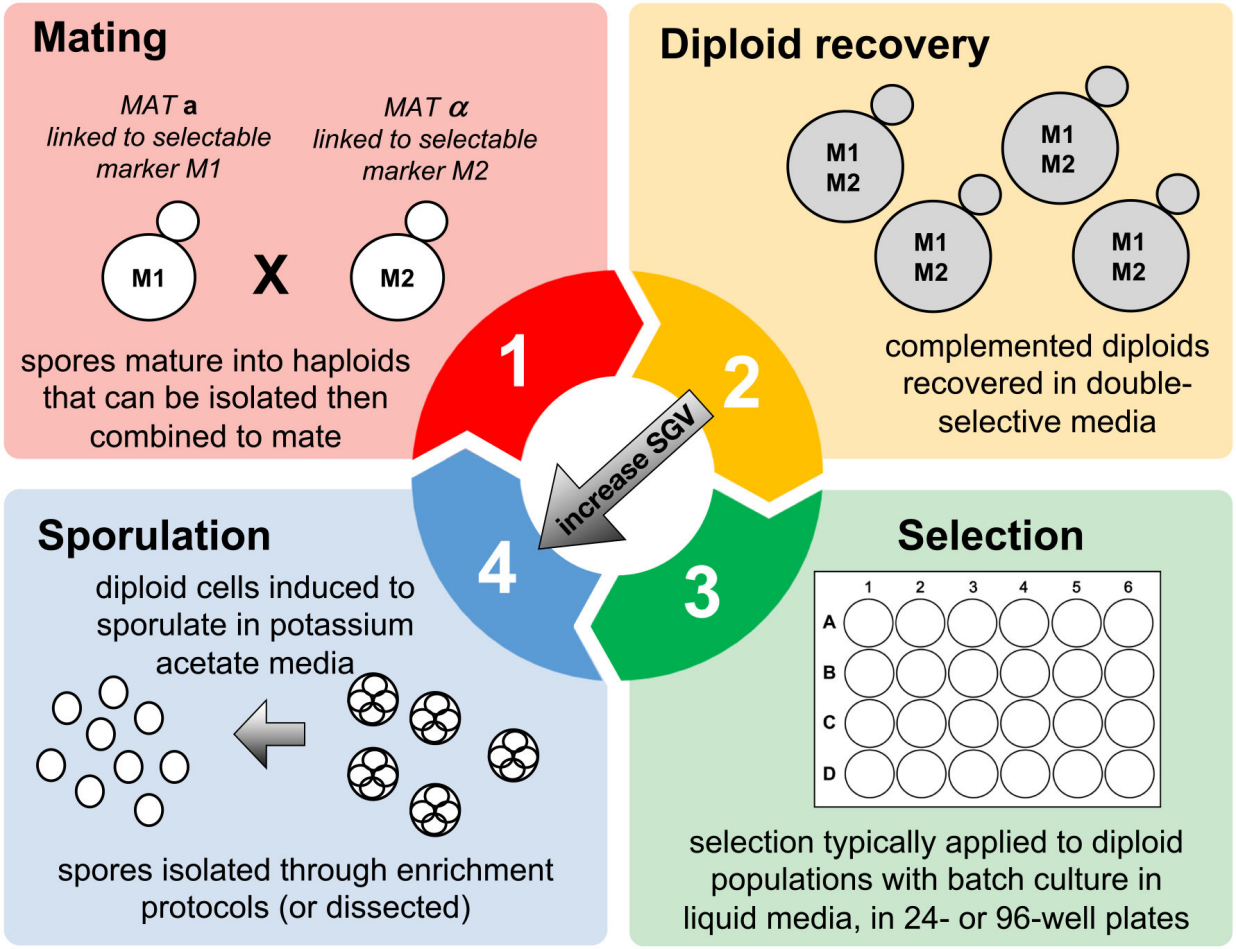
The four general phases of a yeast evolution experiment involving standing genetic variation. First, two or more haploid strains with different genetic backgrounds are crossed, and the products of that cross verified by growth of diploids on selective media. These diploids can be further outcrossed to increase the standing genetic variation in the population (illustrated by the diagonal arrow) or they can be transferred to liquid media for selection. During the selection phase, yeast can reproduce asexually or steps can be taken to periodically induce sexual reproduction. These steps involve transfer to sporulation media, the verification of viable spores, and the enrichment of spores through enzymatic, chemical, and/or mechanical disruption of asci. Isolated spores mature into haploid cells that can again be crossed, starting the cycle anew. All studies listed in Table 1 feature each of these general steps, although not necessarily in the same order, and with significant variation in design parameters

There are four technical phases to think about when designing a yeast evolution experiment with standing genetic variation: (i) generating initial variation through crossing; (ii) recovering recombinant individuals harboring variation; (iii) imposing selection; and (iv) increasing and/or maintaining variation through additional outcrossing (Fig. 1). The studies listed in Table 1 tend to feature each of these phases, although not necessarily in the same order, and with some methodological choices differing among them. For the remainder of this section, I will expand upon the experimental design options to consider in each phase in the order listed above.

### The Initial Cross

Any yeast evolution experiment involving standing genetic variation requires starting with an ancestral population harboring some level of genetic diversity, which is accomplished by crossing two or more strains with different genetic backgrounds. Generally, the more strains that are crossed, the higher diversity in the resulting population; for example, Li et al. (2019) used outbred populations initially made from either 2 or 4 parental (or “founder”) strains and observed approximately twice as many SNPs segregating in the latter populations. But, careful thought should go into any crossing design. Phillips et al. (2021) found that using more founder strains does not necessarily lead to more SNPs segregating in recombinant populations and that in some cases variation will decrease with an increasing number of founders. This inconsistency likely arises from variation in traits related to mating; not all strains have high mating efficiencies and/or mate well with specific other strains, which could lead to losses in spore viability and underrepresentation of a founder genotype. This underrepresentation should become exacerbated with the addition of more strains with low or unknown mating efficiency. So, while it is best to choose founder strains that are very genetically distinct (e.g., belonging to the different major lineages identified by Liti et al. (2009)), it is also advisable to verify that the chosen strains mate effectively prior to starting an experiment. In fact, the best practice involves careful tracking to ensure not only that two strains successfully mate, but consistently produce asci with four viable spores—this can be confirmed by spore dissection (as shown by Phillips et al. 2021).

While recombinant yeast populations can be generated by any researcher as the first step of an experiment, there are several existing “synthetic recombinant” populations that have been explicitly designed to explore the role of standing variation in the evolution and architecture of complex traits. These include the SGRP4X (Cubillos et al. 2013), 4-way cross; 4-way, 8-way, and 12-way crosses described by Phillips et al. (2021); and the 18X populations, made by crossing 18 founders, described by Linder et al. (2020). The Linder et al. (2020) populations are especially well suited for evolution experiments, as they were developed while simultaneously generating de novo genome assemblies of the 18 founder strains such that virtually all variation segregating in the recombinant populations is known. Significant computational resources accompany the biological resources, including software to map haplotype frequencies in any evolved populations derived from the 18X. In evolution experiments with standing genetic variation, tracking haplotype frequencies in addition to SNP frequencies has been shown to increase the power to identify genomic regions underlying adaptive change (e.g., Burke et al. 2014; Linder et al. 2022), as well as to distinguish between models of adaptation (Barghi and Schlötterer 2020). So, it is best practice to design any such experiment so that observed standing variation can be resolved back to the founder genotypes, whether one’s goal is to map individual traits or to analyze adaptive dynamics. To give an illustrative example of how this practice can improve trait mapping, Wing et al. (2020) showed that the signature of adaptive change in an evolution experiment for freeze–thaw tolerance was associated with a single genomic region; in this case a wild North American soil isolate was the only one of the four founders bearing the adaptive haplotype. To give an illustrative example of how this practice improves studies of adaptive dynamics, Linder et al. (2022) showed that across a range of selective environments, adaptation was almost always associated with only one of the 18 founder genotypes, suggesting that selection tends to favor rare haplotypes.

An essential feature of any population one might use to start an evolution experiment with standing variation—whether it be one of the aforementioned resources or a newly synthesized population—is the ability to confirm and track successful mating events. Prior experiments have either used (i) strains engineered such that the different mating types have different auxotrophies, meaning that mated diploids can be recovered in dropout media (e.g., Parts et al. 2011, Burke et al. 2014, Vasquez-Garcia et al. 2017, Li et al. 2019) or (ii) strains engineered such that the different mating types express different drug resistance cassettes, and mated diploids can be recovered in media supplemented with multiple antifungal drugs (e.g., Macdonald et al. 2016, Kolsheleva and Desai 2018, Leu et al. 2020; Linder et al. 2022; Phillips et al. 2022). In either case, haploid strains should first be heterothallic, with the *HO* gene knocked out. And in either case, both the haploid and diploid phases of life history can be controlled in a straightforward way with selective media (favoring *MAT* **a** haploids, favoring *MAT* α haploids, or favoring **a**/α diploids).

### Recovering Recombinant Diploids

After the initial cross, recombinant diploids can be recovered in selective media (typically agar plates) and transferred by scraping to use in the next phase of the experiment. Prior to a long-term evolution experiment, additional rounds of outcrossing may be imposed in order to further shuffle the genetic variation within the population. If this is the goal, these recovered diploids should be transferred to sporulation-inducing media such that spores can be recovered and haploid cells isolated and mated again in series (represented by the diagonal arrow in Fig. 1). In the context of an ongoing evolution experiment, these diploids will likely be transferred to the medium/environment that involves the selective regime. This step of diploid recovery is when effective population size (*N*_*e*_) can be best assessed. Because diploid recovery is usually achieved on solid medium—complemented diploids become evident as CFU counts on agar plates either lacking or supplemented with selective agents—CFUs are an accurate estimate of the number of breeding individuals in the population. Ideally, this number should be tracked in every replicate population, in every cycle of the experiment. This allows investigators to monitor population size and discard any replicates in which population size falls too low. *N*_*e*_ is harder to track in evolution experiments with standing genetic variation, compared to clonal experiments, because in clonal experiments census population size (easily assessed by absorbance in liquid media) is equal to the effective population size. *N*_*e*_ is also important to track in experiments with standing variation because bottlenecks that reduce the population size too severely will influence evolutionary dynamics in undesirable ways; in other words, the effects of drift will outweigh the effects of natural selection on the standing variation. Most experiments in the literature have reported a *N*_*e*_ threshold of > 10^5^.

There is additional value in tracking the number of diploid CFUs at this step as it allows for a more accurate estimate of the number of generations elapsing in the experiment. It is typical and straightforward to track the number of “competitive” cell doublings occurring in liquid media with absorbance and using before and after values to calculate the number of asexual generations that have elapsed during a given growth period. While this is often the most meaningful number of generations to report for a given experiment (see the “Selection” section), a considerable number of cell divisions will also occur on solid media during the diploid recovery step. Estimating the number of “noncompetitive” asexual generations at this step is more challenging but can be done by comparing the effective population size to the census size. For example, the observed CFU values—representing the number of unique individual cells that initiated the plate phase—can be compared to the total number of cells on the plate (this can be estimated by scraping the cells into liquid media and measuring absorbance of the cell suspension). When transferring diploid cells to the next experimental phase, ideally both census size and effective size will be standardized across replicate populations. Of course in practice, such tracking can quickly become laborious with an increasing number of experimental replicates. Concessions can be made in the interest of throughput, such as tracking a subset of replicates or choosing only to standardize census population size during transfers. But, generally it is worth the extra time and effort taken to optimize and standardize *N_e_*, as even when this care is taken, considerable variation in *N*_*e*_ has been observed across replicates. In the experiment carried out by Burke et al. (2014), analysis of sequence data pointed to 5/12 experimental replicates experiencing serious bottlenecks in at least one sequenced timepoint such that follow-up studies (e.g., Iranmehr et al. 2017; Vlachos et al. 2019; Phillips et al. 2020) excluded these replicates entirely. A related recommendation is to increase the number of experimental replicates in anticipation of this type of downstream data curation.

### Selection

The most common time to impose a selection regime in this type of evolution experiment is immediately after recombinant diploids have been obtained by recovery on selective media. Typically, this is achieved by adding a chemical to liquid media and initiating batch culture of diploids (although sometimes the haploids are cultured separately selective environments, e.g., McDonald et al. 2016; Leu et al. 2020, and Kolsheleva et al. 2018 who use both methods). Cultures can then be diluted back at specific intervals, based upon the desired threshold census size and the severity of the selective agent. In experiments involving rich media and selective agents that do not dramatically slow cell growth, this tends to involve a 1:10^3^ dilution after 24 h (e.g., Burke et al. 2014). In experiments with stronger selection, less aggressive sampling and/or longer growth phases will increase the number of competitive (asexual) generations per unit time, which is generally desirable. For example, Phillips et al. (2022) maintained experimental populations in media supplemented with ethanol for 48 h, with a 1:10^2^ dilution midway through, on a weekly basis. Linder et al. (2022), which implemented a variety of stressors, including chemicals that dramatically slowed growth rate, diluted cultures by 1:10 every 24 h for three days, also on a weekly basis. Culture vessels and volumes are additionally important choices when considering how to maximize cell turnover in an experiment; in the Burke lab, we generally maintain cultures in total volumes of 1 mL in individual wells of 24-well plates. We find that these choices serve to (i) maintain populations at large enough census sizes to prevent unwanted bottlenecks; (ii) maintain sufficient spatial separation between cultures to avoid unwanted cross-contamination (we use every other well of the plate and include sterile media in alternate wells to track the rates of contamination events); and (iii) maintain sufficient aeration in each well, given their relatively large surface area, which prevents unwanted cell clumping. Other labs will make choices that serve their goals best; for example, Linder et al. (2022) and others have opted to maintain populations in 96-well deep-well plates to increase experimental throughput, aided by the use of liquid-handling robots. Notably, these authors reported evidence of such significant clumping in their experimental populations, perhaps as a result of the choice to use 96-well plates, that phenotyping of evolved populations became difficult. In general, there is no “one size fits all” protocol to recommend; when designing an experiment, individual investigators should carefully consider how their specific hypotheses might be impacted by these different experimental parameters and develop protocols that are tailored to best suit their priorities.

Regardless of the protocol used, it is essential to survey population size by measuring cell density at all transfer points (e.g., dilutions of cultures in selective media, transfers of cultures from selective to sporulation media, cultures before and after sporulation, and mating) to keep track of the approximate number of cell doublings taking place in each phase and to keep a meaningful record of the evolutionary timescale of the experiment. The selection phase, involving dilutions to increase generational turnover, can continue for as long as an investigator wishes. In experiments that do not actively maintain the maintenance of genetic variation via outcrossing, this typically lasts for a few hundred asexual generations or until an extreme phenotype is observed in the evolved populations relative to the ancestor. Alternatively, experiments that incorporate additional outcrossing to shuffle genotypes will “pause” selection to induce a cycle of sporulation and mating and then re-instate the selective regime. While individual studies vary in the frequency and timing of this back and forth, depending on the research questions being addressed, at least 10 generations of selection should elapse prior to an additional outcrossinging event. The outcrossing process itself involves significant stresses that lead to adaptation even in the absence of any other selective agent (e.g., Burke et al. 2014), so too few generations of selection could result in a noisy or otherwise obscured signal of change.

In the design of any selective regime, a number of experimental parameters can be optimized to reduce potential noise that may present at the end of the experiment, such as an unambiguous signal of genomic change. In this author’s view, population replication is the most important of these. Virtually all evolution experiments that start with standing genetic variation demonstrate that adaptation is repeatable at the genotype level; in other words, the same genomic regions respond to change across multiple replicate populations. This is both an important evolutionary discovery and notable from an analytical perspective. Individual alleles that respond to selection in a non-parallel way can be identified by scrutinizing replicate populations separately (e.g., for evidence of different de novo mutations). But identifying regions that change in parallel across multiple replicates requires a large number of such replicates to achieve statistical power. The experiment by Burke et al. (2014) involved 12 replicate populations, and applying linear regression to genome-wide allele frequency changes over time revealed five candidate regions of strong statistical association. When the authors randomly downsampled their dataset to only include five replicates, they identified no candidate regions at all. Possibly as a result of this empirical power analysis, subsequent experiments of this type usually involve 12 or more replicates. As mentioned in the previous section, high replication also provides a “buffer” against experimental errors, such as unintentional bottlenecks or contamination, and especially when liquid-handling robots are used for transfers, do not add significant time or labor costs to the experiment itself.

Other experimental parameters to consider during the selection phase include experimental duration, timepoint sampling, and the use of a control treatment. Regarding experimental duration, generally a longer experiment results in a greater signature of adaptive change, as measured by phenotypes and genotypes evaluated over time. Phillips et al. (2020) consider the role of timepoint sampling and report that when the goal of the study is trait mapping, genome sequencing at the beginning and end of the experiment is sufficient to pinpoint regions associated with the focal trait. But, repeated sampling is required when the goal of an experiment is to analyze evolutionary dynamics, such as to describe the trajectories of adaptive alleles. With respect to a control treatment, the literature is varied, but it is becoming clearer that even the non-selective phases of experiments exert evolutionary forces on populations, such that investigators would arrive at very different conclusions about the fate of standing genetic variation in evolved populations if a control treatment had not been used (Phillips et al. 2022). Thus, including a control treatment is a recommended best practice, though the full extent of how these controls impact our depth of understanding of experimental results is an active topic of research.

### Increasing and/or Maintaining Variation

Once a population has been identified as an appropriate ancestor for laboratory evolution, a critical experimental choice is whether to continue to induce outcrossing as selection proceeds. The simplest design involves sampling the desired ancestral population to initiate a number of experimental replicates and imposing a selective regime for a designated period of time, while the populations evolve asexually. With this design, there are millions of unique clonal lineages competing in the population and those with the most beneficial combination of alleles will eventually dominate. With genome sequencing, these beneficial haplotypes can be revealed by scans of nucleotide diversity (to detect evidence of recent selection) and/or comparing allele frequencies in evolved populations to those in the ancestor. This approach has been used to dissect the genetic basis of several traits, including thermal stress (Parts et al. 2011), resistance to anticancer drugs (Vasquez-Garcia et al. 2017, Li et al. 2019), freeze–thaw tolerance (Wing et al. 2020), and stress imposed by various chemicals (Ament-Velásquez et al. 2022). This type of evolution experiment typically results in fairly rapid divergence from the ancestral population such that specific genomic regions can be pinpointed as harboring candidate variants underlying adaptation within a few hundred asexual generations. Indeed this method shares many similarities with QTL mapping approaches that have long been employed in yeast, including bulk segregant analysis and X-QTL (e.g., Ehrenreich et al. 2010; reviewed by Liti and Louis 2012). In fact, any of the entries in Table 1 with “asexual” as the mode of reproduction can appropriately be considered QTL mapping experiments, as well as evolution experiments.

One downside of the choice to prevent additional outcrossing in an experimentally evolving population is that in its absence, given enough time, a single clonal lineage will outcompete all others—essentially, clonal interference will lead to clonal exclusion. An additional consideration is that the absence of sex will result in an inability to uncouple beneficial variants from potentially deleterious hitchhiking alleles; this complicates evolutionary dynamics and generally leads to a slower and less efficient adaptive process (e.g., Macdonald et al. 2016). By contrast, imposing regular outcrossing as part of a selective regime leads to the continuous shuffling of genetic variation via recombination and a population capable of purging deleterious alleles or combinations of alleles. This type of design has generally been more popular with investigators whose stated interests lie more in dissecting general features of adaptive dynamics (e.g., Burke et al. 2014; Phillips et al. 2020, 2022), or the consequences of sexual reproduction on adaptive dynamics (e.g., Macdonald et al. 2016, Kolsheleva and Desai 2018, Leu et al. 2020), than on trait mapping. Of course in theory this latter type of design can accomplish these goals simultaneously (*cf*. Linder et al. 2022), although the inclusion of regular outcrossing into a selection regime significantly complicates all aspects of the experiment, from benchwork to data analysis. So, a general recommendation emerging from these studies is that continued outcrossing is not required when the investigator’s goal is to dissect the genetic basis of specific traits, but it is a desirable feature of any experiment where the goals are more general, pertaining to fundamental evolutionary questions.

In any yeast population with standing genetic variation, that variation is initially created then maintained by the twin engines of mating and sporulation. Having already discussed mating and recovery of recombinant diploids, I now turn my attention to sporulation. Yeast biologists that work with a variety of strains (particularly those not commonly used in lab settings) know that there is a huge amount of variation in sporulation efficiency among them and that a number of sporulation conditions can be optimized to increase sporulation efficiency (e.g., Elrod et al. 2009). The genetic background of a strain, the composition of the sporulation media, and the density and volume of the culture (assuming sporulation occurs in liquid) all significantly contribute to the timing and completeness of sporulation (cf. Dunham et al. 2015). So, for yeast evolution experiments with standing genetic variation, optimizing sporulation efficiency in the ancestral population is a key step. This can be done by deliberately choosing founder strains with known high sporulation efficiencies (e.g., Cubillos et al. 2013) and/or taking a starting population through multiple rounds of mating and sporulation, which should lead to the evolution of increased sporulation efficiency (as reported by Phillips et al. 2021). This is yet another reason why using a pre-existing community resource (e.g., the SGRP4X or 18X, available upon request from their respective labs of origin) is strongly recommended for future evolution experiments. Ultimately, it is valuable to work with a population that is known to sporulate with high efficiency (75–100%) within a short period (2–3 days), in the small volumes of a culture plate well (typically 1 mL of 1% potassium acetate media).

In order to maintain standing genetic variation in a yeast population, cells not only need to sporulate, they must also outcross. Promoting outcrossing involves taking steps to ensure that yeast asci, containing four recombinant haploid spores, are broken so that intra-ascus mating (i.e., selfing) cannot occur. Most protocols for spore enrichment (e.g., Rockmill et al. 1991) can accomplish this goal and can be easily modified to fit the routine and throughput of an evolution experiment. While investigators have adapted spore enrichment protocols in a variety of ways, all of the studies in Table 1 use Zymolyase (Zymo Research) to digest ascus walls and free spores. Then, vegetative diploids must be eliminated from the population, as any cells that do not sporulate continue to divide clonally and therefore threaten the maintenance of genetic variation. This goal is typically accomplished either by isolating haploids with selective media, on which diploids cannot grow (e.g., Macdonald et al. 2016, Kolsheleva and Desai 2018, Leu et al. 2020), or by exposing the mixed cultures to chemicals that kill diploids such that only the spores survive. While ether has long been known to serve this purpose (Bahalul et al. 2010), others are finding success with commercial protein extraction reagents, such as Y-PER (Thermo). In addition to these reagents, some investigators are also implementing mechanical agitation by shaking with glass beads (e.g., Burke et al. 2014, Linder 2020, Linder et al. 2022, Phillips et al. 2022) which goes even further to weaken ascus walls and eliminate vegetative cells. And, Burke et al. (2020) demonstrate that adding a brief heat shock step after sporulation is sufficient to kill unsporulated diploids, but not spores. While each of these measures (chemical, mechanical, thermal) is recommended to enrich spores from a mixed culture, using all three together can come at the cost of some spore viability, especially in particular strain backgrounds (as shown by Phillips et al. 2021). So, some optimization is needed here too, to balance the negative consequences of incomplete spore enrichment—which include the “cheating” of asexual genotypes and the reduction of outcrossing, against the negative consequences of too much spore enrichment, which include spore death and unwanted bottlenecking.

A feature in common to all the existing populations described in the “The Initial Cross” section is that they have already been through 12 rounds of outcrossing. While the choice of 12 is somewhat arbitrary (it likely emerged as the convention because this was the choice established by Cubillos et al. 2013), it has become clear that these additional rounds of outcrossing are very valuable for any downstream experimental evolution work. Given that crossing divergent founders can be challenging, significant sorting of standing variation occurs during these initial cycles, despite no other selective agent being applied. In other words, it is helpful to “pre-adapt” a yeast population with standing genetic variation to the laboratory protocols necessary for inducing outcrossing, prior to using it in an evolution experiment. Investigators have repeatedly shown that these protocols impose selection on their own. Cubillos et al. (2013) identified signatures of selection associated with these protocols and identified candidate regions underlying mating and sporulation efficiency. Phillips et al. (2021) did the same and noted that even when starting with the exact same four founder strains, these signatures of selection did not necessarily overlap. This introduces the additional complexity that every investigator’s hands are different and that even implementing the same or similar protocols in different laboratory environments could lead to different patterns of standing variation as a result. In fact, Burke et al. (2014) obtained the SGRP4X population of Cubillos et al. (2013) and imposed an additional 18 cycles of outcrossing on this population in an independent laboratory. The result was a completely distinct signature of selection than that reported by the predecessor. This result reinforces the idea that the outcrossing protocols themselves are stressful and impose selection, and it also suggests that no two outcrossing protocols are likely to impose the exact same selective pressures. For these reasons, it is ideal to use a pre-developed population as the ancestor for an evolution experiment *and also* impose several rounds of any new outcrossing protocol in advance of adding a selective regime. As previously discussed, including a control treatment that consists exclusively of protocols related to outcrossing, handled in parallel with treatments involving both outcrossing and a specific selective agent, provides the most comprehensive design.

## Unique Challenges Facing Experiments with Standing Genetic Variation

The majority of yeast evolution experiments do not prioritize the maintenance of standing genetic variation and for good reasons. Including variation complicates every level of experimental design, from the choice of the ancestral population to the numerous steps required to maintain variation and to the ultimate analysis of the experimental data.

Considering the genomic analysis of these experiments provides one platform for discussing this complexity. While there are many similarities in methods in the genomic analysis of data from experiments with vs. without standing genetic variation, there are important differences to keep in mind (see Martinez and Lang 2023) for an overview of genomic analysis of experiments featuring initially isogenic yeast populations). Generally, experiments with standing genetic variation involve sequencing entire experimental populations, rather than isolated clones; this so-called Pool-SEQ method (Schlötterer et al. 2015) is common in evolution experiments with non-microbial systems, and most Pool-SEQ best practices have emerged from work with *Drosophila* (e.g., Vlachos et al. 2019). In practice, applying the lessons of *Drosophila* Pool-SEQ to yeast is fairly straightforward. For example, Pool-SEQ requires sequencing large pools (> 100) of individuals to accurately sample the population, but this is trivial in yeast where 1 mL can harbor hundreds of millions of cells. Pool-SEQ also requires deep sequencing coverage (> 50X) of individual samples, as coverage serves as the denominator for the ascertained allele frequency estimates. While this places considerable constraints on experiments with *Drosophila* due to cost, the small genomes of yeast make this achievable with multiplexing. As an example, my lab routinely combines 48 uniquely barcoded samples for Illumina sequencing, and one PE150 sequencing lane of such a multiplexed library usually returns acceptably high coverage per sample. Evenness of coverage across samples is equally important as exceeding a coverage minimum, and this can be solved experimentally (by re-sequencing individual barcoded libraries) or computationally (by scaling, *cf.* Wiberg et al. 2017). While Pool-SEQ methods continue to become more standardized and refined, my lab uses a general pipeline whereby the variant caller GATK (Van der Auwera & O’Connor 2020) uses BWA-MEM (Li 2013) to align raw data to the *S. cerevisiae* reference genome and create a VCF file for all variants identified across all populations. The VCF file can be used as an input for tools that predict the functional effects of individual SNPs, such as SNPEff (Cingolani et al. 2012). This VCF file can also be converted into a “raw” SNP frequency table by extracting the AD (allele depth) and DP (unfiltered depth) fields for all SNPs passing quality filters; the former field being used as the allele count of the presumed SNP (non-reference allele) and the latter used as the total coverage observed at the site. The raw SNP table is then a useful format for data sharing, as it is amenable to many analysis strategies depending upon the research question being asked. As an example, this SNP table can be used to estimate haplotype frequencies in evolved populations, a method discussed in the “The Initial Cross” section, provided that the sequences of the founding strains are known—and most are publicly available (Peter et al. 2018).

Once a candidate genetic variant is identified as potentially associated with an evolved phenotype (because it increased in frequency over time), it is not clear how to best validate the functional consequences of that variant. While it is technically straightforward to swap one allele for another in yeast, it is not easy to do this simultaneously in the numerous and diverse genotypes present in an outbred population. One could potentially isolate a number of clones from the ancestral population, achieve allele swaps in these, and use competition experiments to determine whether a variant confers a selective advantage in a specific environment, relative to the “wild type” allele. But, with the thousands of haplotypes present in the ancestral population, the allele in question must have a very large effect and/or a huge number of clones would need to be evaluated in this way, in order to arrive at a convincing conclusion. So, no evolution experiment with standing genetic variation has attempted functional validation at the time of writing this article, although I expect this will be an area of growth for the field in the years ahead.

Another unique challenge facing these specialized experiments is the complicated life history sequence that the yeast must go through. In a typical experimental protocol that includes all steps listed in Fig. 1, yeast cells must successfully mate in one media type, recover in another media type, sporulate in a third media type, and survive a number of stressors associated with spore enrichment, all before experiencing the presumably stressful conditions of the selective environment. Such a protocol virtually guarantees that the focal trait under selection—the one of primary interest to the investigator—is just one of many features of a complex environment. Such a complex environment inevitably will lead to the selection of “cheater” genotypes, perhaps haploid cells that can survive selective conditions intended for complemented diploids and/or diploid cells that can escape the steps intended to induce sporulation and mating. Linder et al. (2022) reported significant evidence of such asexual cheater-type cells, the instances of which were positively correlated with the intensity of the main selective agent. While they acknowledge that the emergence of cheater genotypes is itself an interesting observation worthy of further study, they could restrict their analysis to populations with no evidence of cheating, due to the high levels of replication in the experiment. So, some ability to monitor and report evidence of cheating is necessary in experiments like these.

Outside of the possibility of cheating, the complex life history brings up the pertinent question of what should be considered a generation in these experiments. The studies listed in Table 1 generally focus on the total number of asexual generations in the experiment, but doing this limits our ability to generalize conclusions to other eukaryotic species in which a generation can only be the result of sex. Some of these very studies (e.g., Kolsheleva and Desai 2018) show that the number of asexual generations in between sexual cycles impacts adaptive dynamics, suggesting that considering asexual generations alone is naïve. On the other hand, the recombination rate is sufficiently high in *S. cerevisiae (cf*. Liu et al. 2019) such that a single instance of outcrossing likely shuffles the genome to a much higher degree than it does in obligately sexual organisms. Selection is thought to leave footprints of reduced variation roughly equal to one-tenth of the selection on the allele in units of recombination (Kim and Stephan 2002). If yeast were obligately sexual and recombination occurred every generation, a selective sweep associated with a selection coefficient of 0.01 would leave a 0.1-cM footprint in the genome. So, in an experimental design where recombination occurs only every ~ 10 generations (the minimum recommended in the “Selection” section), the size of a selection footprint would be something like 10*0.1 = 1 cM. In *S. cerevisiae*, 1 cM averages about 3 kb (Saccharomyces Genome Database), so in this situation, the signature of selection could be localized to a small region ~ 3 kb in size. In practice, this is in fact what has been observed; for example, with a design that involved sexual cycles every ~ 30 asexual generations, Burke et al. (2014) observed their strongest candidate genomic regions spanning < 10 kb. Such findings support the idea that while the yeast life history involved is complex, experimental results are as good or better (in terms of the genomic resolution of candidate regions) than similar work in obligately sexual species.

## Conclusion

What have we learned from these yeast evolution experiments that tackle the complexity of sexual recombination and standing genetic variation? They reveal that adaptation from standing genetic variation is rapid, often parallel across independent populations, and is made more efficient by sexual reproduction. These studies repeatedly demonstrate that de novo beneficial mutations are not the primary drivers of adaptation, as standing variants respond first to natural selection. This outcome is notable because yeast is currently the best microbial (i.e., strong inference) system capable of addressing the impacts of both sources of variation. This observation also applies to structural variation in the genome; copy number variants and large-scale chromosomal mutations do not appear to be major drivers of adaptation in these experiments. As aneuploidy, and/or an increase in overall ploidy, is a common outcome of traditional yeast evolution experiments, this observation serves to further establish experiments with standing genetic variation as distinct from those without. I argue that the methods described in this article are now well established, and as a result, yeast has emerged as an appropriate and valuable model for the empirical study of polygenic adaptation.

## Figures and Tables

**Table 1:** List of yeast evolution experiments featured in this review

Study	Selection regime	Founder strains	Experimental replicates	Sexual or asexual	Major results
Parts et al. 2011	Thermal stress	2	2	Asexual	Thermal stress QTL mapped with high resolution; candidate variants plateaued in frequency without fixing
Cubillos et al. 2013	Thermal stress, arsenite, paraquat	4	2	Asexual	Stress resistance mapped to genomic regions with high resolution, including at the haplotype level; SGRP4X resource established
Burke et al. 2014	Batch culture in rich media	4	12	Sexual	SGV drives adaptation in sexual populations; > five-fold replication needed for strong inference
McDonald & Desai 2016	Batch culture in rich media	1	6 sexual, 6 asexual	Both	Sex speeds adaptation by breaking Muller’s ratchet
Vásquez-Garcia et al. 2017	Hydroxyurea, rapamycin	2	6 or 8 per treatment	Asexual	SGV, de novo mutations, and genome instability all significant in the evolution of drug resistance
Kolsheleva et al. 2018	Batch culture in rich media	2	12 “frequent sex,” 12 “rare sex,” 12 asexual	Both	Increasing recombination increases the effectiveness of selection
Li et al. 2019	Hydroxyurea, rapamycin	4	8 per drug	Asexual	More initial SGV leads to more QTL and increases complexity of trait architecture
Leu et al. 2020	Thermal and NaCl stress	1	6 sexual, 6 asexual	Both	Sex facilitates adaptation to dynamic environments
Wing et al. 2020	Freeze/thaw stress	4	12	Asexual	A single freeze–thaw stress QTL mapped; some SGV is lost during cryopreservation
Linder et al. 2022	16 chemical stressors	18	variable per treatment	Both	Adaptation is driven by selection on rare variants; many populations evolved “cheater” strategies that avoided sex
Ament-Velásquez et al. 2022	Ethanol, salt, lithium acetate	2	4 or 5 per treatment	Asexual	Asexual adaptation driven by both SGV and de novo mutations; less parallelism observed in treatments with stronger selection
Phillips et al. 2022	Ethanol	12	20 per treatment	Sexual	Distinct adaptive responses observed in treatments with different selection intensities

These studies are distinct from traditional yeast evolution experiments in that they either begin with ancestral populations that harbor standing genetic variation and/or they impose sexual cycles to shuffle genetic variation as the experiment proceeds
